# Differential Skewing of Circulating MR1-Restricted and γδ T Cells in Human Psoriasis Vulgaris

**DOI:** 10.3389/fimmu.2020.572924

**Published:** 2020-12-03

**Authors:** Vera Plužarić, Mario Štefanić, Martina Mihalj, Maja Tolušić Levak, Ivanka Muršić, Ljubica Glavaš-Obrovac, Martin Petrek, Peter Balogh, Stana Tokić

**Affiliations:** ^1^ Department of Medical Chemistry, Biochemistry and Clinical Chemistry, Faculty of Medicine, University of Osijek, Osijek, Croatia; ^2^ Department of Dermatology and Venerology, University Hospital Osijek, Osijek, Croatia; ^3^ Department of Nuclear Medicine and Oncology, Faculty of Medicine, University of Osijek, Osijek, Croatia; ^4^ Department of Physiology and Immunology, Faculty of Medicine, University of Osijek, Osijek, Croatia; ^5^ Department of Histology and Embryology, Faculty of Medicine, University of Osijek, Osijek, Croatia; ^6^ Department of Pathological Physiology, Faculty of Medicine and Dentistry, Palacký University, Olomouc, Czechia; ^7^ Department of Immunology and Biotechnology, Faculty of Medicine, University of Pecs, Pecs, Hungary

**Keywords:** psoriasis, cytokines, gammadelta T lymphocytes, mucosal associated invariant T cells, MR1

## Abstract

Psoriasis vulgaris (PV) is a chronic, recurrent inflammatory dermatosis mediated by aberrantly activated immune cells. The role of the innate-like T cells, particularly gammadelta T (γδT) cells and MR1-restricted T lymphocytes, is incompletely explored, mainly through animal models, or by use of surrogate lineage markers, respectively. Here, we used case-control settings, multiparameter flow cytometry, 5-OP-RU-loaded MR1-tetramers, Luminex technology and targeted qRT-PCR to dissect the cellular and transcriptional landscape of γδ and MR1-restricted blood T cells in untreated PV cases (n=21, 22 matched controls). High interpersonal differences in cell composition were observed, fueling transcriptional variability at healthy baseline. A minor subset of canonical CD4^+^CD8^+^MR1-tet^+^TCRVα7.2^+^ and CD4^+^CD8^-^MR1-tet^+^TCRVα7.2^+^ T cells was the most significantly underrepresented community in male PV individuals, whereas Vδ2^+^ γδ T cells expressing high levels of TCR and Vδ1^-^δ2^-^ γδ T cells expressing intermediate levels of TCR were selectively enriched in affected males, partly reflecting disease severity. Our findings highlight a formerly unappreciated skewing of human circulating MAIT and γδ cytomes during PV, and reveal their compositional changes in relation to sex, CMV exposure, serum cytokine content, BMI, and inflammatory burden. Complementing numerical alterations, we finally show that flow-sorted, MAIT and γδ populations exhibit divergent transcriptional changes in mild type I psoriasis, consisting of differential bulk expression for signatures of cytotoxicity/type-1 immunity (*EOMES, RUNX3, IL18R*), type-3 immunity (*RORC*, *CCR6*), and T cell innateness (*ZBTB16*).

## Introduction

Psoriasis is a common and diverse, but poorly understood autoinflammatory dermatosis affecting up to 3% of the Caucasian population. Plaque-type (vulgar) psoriasis (PV) comprises most cases, but other forms have been also described (1). Once manifest, it is typically a relapsing disease often associated with systemic manifestations and comorbidities. The etiology is not clear, but genetic predisposition, in addition to microbial dysbiosis, dietary factors, and immune response, can trigger the disease (2).

On the molecular level, a range of resident and recirculating TNFα- and IL-17A-producing cells instructs the development of aberrant skin inflammation (3), but understanding how this diversity fits into psoriasiform inflammation is still limited. Consequently, the precise composition of blood and lesional cells in PV remains unclear and almost certainly varies with different pathologic settings.

In humans, a variety of IL-17-producing CD4^+^ and CD8^+^, conventional (4–6) and innate-like (γδ (7–10)), mucosal associated invariant T (MAIT) (11) and invariant natural killer (iNKT) (12)) T cells, is enriched in psoriatic skin. The latter ones, particularly semi-invariant γδ T cells, are central to dermal integrity and repair (13), and represent the principal IL-17 source in several animal models of skin inflammation and PV (8, 14–18). Their human counterparts differ in number, distribution, and T-cell receptor (TCR) repertoire (19), and are often delineated into Vδ2^+^ and Vδ2^-^ subsets (20). Vδ2^+^ cells largely dominate peripheral adult blood, often co-express Vγ9 chain and mount prototypic anti-microbial innate immune responses. The Vγ9Vδ2 population, in particular, delineates an important pro-inflammatory, skin-homing γδT cell compartment in PV (10). Conversely, Vδ2^-^ T cells, particularly Vδ1^+^ T cell compartment, are mostly confined to epithelial layers and mucosal surfaces, exhibiting clonally expanded TCR repertoires (21). In addition, different subsets of circulating γδ T cells can be discriminated in healthy individuals based on CD3 and/or TCR expression levels: a larger subset of γδ T cells expressing intermediate levels of TCR (hereafter CD3^+^γδTCR^int^), and a smaller fraction of γδ T cells expressing high levels of TCR (CD3^+^γδTCR^high^), the latter containing IL-17^+^δ1/δ3^+^ effectors (22, 23), but no data regarding the variance between these γδ T cell subsets currently exists for PV. Complementing these observations, distinct gene co-expression networks have been associated with the functional heterogeneity, TCRδ usage, and cell-type specification of γδT cells, but the transcriptional landscape of the human γδ T cell lineage in PV remains mostly uncharacterized.

Similarly, even less is known, about the MAIT population, another major innate-like T cell subset in humans. Evidence suggests that these cells traffic extensively (24) and may contribute significantly to IL-17 production in a highly inflammatory environment (25) by exerting rapid and direct effector responses prior to and independently of the TCR signaling (26). Under homeostatic conditions, the majority of the CD8^+^IL-17^+^ T cell population in blood belongs to MAIT cell (27), but how these cells are distributed in PV remains unclear. Much alike to γδ T cells, MAIT cells express a high level of surrogate markers, such as CD161 and IL-18Rα, and rely on semi-invariant TCR (Vα7.2) which recognizes microbial riboflavin and folate metabolites bound on major histocompatibility complex class I-related protein-1 (MR1) (26, 28–30). Consistent with this, MAIT and γδ T cells also exhibit close similarity in their transcriptional nature, and share analogous effector subsets (31). At present, insights into the regulation of human γδ and MAIT cells in PV remain scarce (32, 33) and are mainly fueled by findings obtained in mouse models (8, 14, 16, 18) or by use of CD161 and TCRVα7.2 as surrogate markers of MAIT cells (11). A single study is available on dermal MAIT cells (11), describing similar ratios of CD8^+^CD161^+^TCRVα7.2^+^ cells in healthy and psoriatic human skin; however, CD4^-^CD8^-^, CD4^+^, and CD4^+^CD8^+^ MAIT cells also exist but have not been probed yet. In addition, the prevalence and phenotype of circulating MR1-restricted T cells, encompassing not only the canonical TRAV1-2^+^MAIT set but also a broader class of atypical TRAV1-2^-^MR1-reactive T cells, are even less constrained (34, 35).

Here, we exploit the advantages of MR1-Ag tetramers to provide unbiased estimates of MR1-restricted T cells and their peripheral blood numbers in PV across a range of cell subsets, thus obviating the need for surrogate markers. Next, we refine the data on blood γδ T cells and their various fractions in relation to disease severity, circulating signature cytokines, and trafficking mediators by using flow cytometry in a cohort of therapeutically-naïve, well-defined PV cases, and their matched healthy controls. In the last step, we perform a targeted gene expression analysis of purified MAIT and γδ blood T cells, and generate their respective transcriptomic profiles in relation to health and PV.

## Materials and Methods

### Study Design and Subject Selection

Twenty-two healthy controls (16 males, 6 females; 23–54 years of age, median age 32 years) and 21 clinically active, well-characterized psoriatic patients (14 males, 7 females; 19–49 years of age, median age 33 years) were recruited at the Department of Dermatology and Venereology of the Osijek University Hospital, Croatia. All participants were unrelated and had no history of impaired hepatic or renal function. Psoriasis vulgaris was defined according to the pathohistological findings of a skin biopsy, and disease severity estimated by the treating dermatologist using Psoriasis Area and Severity Index (PASI) and the Dermatological Life Quality Index (DLQI) questionnaires (36, 37). A complete blood count (CBC) encompassing red blood cell (RBC), white blood cell (WBC), platelet count, WBC differential, hemoglobin, and hematocrit measurements, together with C-reactive protein (CRP) serum levels and erythrocyte sedimentation rate (ESR) were performed in the hospital central laboratory. The body mass index (BMI) and markers of bacterial and viral burden including Mycobacterium tuberculosis (QuantiFERON-TB Gold test), cytomegalovirus (anti-CMV IgG, anti-CMV IgM), hepatitis B (anti-HBsAg) and hepatitis C (anti-HCV) antibody titre, were assessed on the same day, respectively. Patients undergoing systemic immunomodulatory, PUVA (psoralen and ultraviolet A) or nbUVB (narrow band UVB) phototherapy, with autoimmune, malignant and infectious comorbidities or allergic reactions within 6 weeks prior to the testing, were excluded from the study. Written informed consent was collected from all participants prior to the testing, and the study protocol was reviewed and approved by the ethical committee of the Osijek University Hospital (number: R2-9042/2018) and the Faculty of Medicine in Osijek (number: 2158-61-07-18-135).

### Peripheral Blood Mononuclear Cell Isolation, Storage, and Thawing

Peripheral blood mononuclear cells (PBMCs) were isolated from 10 mL of freshly collected, heparinized blood samples fractionated during 25 min at 800g gradient density centrifugation on Lymphoprep medium (STEMCELL Technologies; Germany). Harvested mononuclear cell layer was transferred to a sterile conical tube, topped with PBS buffer up to the 14 mL mark and pelleted by centrifugation at 550g for 10 min. Washing step was repeated once more. Collected PBMCs were gently resuspended in 5 ml of 1x PBS and their cell count and viability were determined with the use of the Countess II automated cell counter (Thermo Fisher Scientific, USA). Following final 5 min centrifugation at 550g, 1x10e6 cells were resuspended in 1 ml of 1xPBS buffer and used immediately in downstream applications. The remaining PBMC collection was counted, pelleted and cryopreserved in 3x10e6 cell aliquots suspended in 0.5 ml of cold FBS (Biosera, France), and an equal volume of pre-chilled (4°C) freezing medium [FBS + 20% of DMSO (AppliChem)] added dropwise. Cryovials were placed in a styrofoam container and stored shortly (24–72h) at −80°C, before being transferred into liquid nitrogen tank.

For staining, cryopreserved PBMCs were thawed rapidly (60s) in a 37°C water bath, transferred into sterile 15 mL tube and dropwise diluted with 10 mL of pre-warmed, supplemented RPMI-1640 culture medium [10% FBS, 1% Na-pyruvate, 0.01M HEPES, Sigma-Aldrich]. Thawed cells were pelleted at 350g for 10 min, suspended in 5 mL of RPMI-1640 buffer, and shortly rested (30 min at 37°C in a CO_2_ water jacketed incubator) before being washed and resuspended in cell staining buffer for the respective experiments.

### Flow Cytometry Analysis of Peripheral MR1-Restricted and γδT Cells

PBMCs aliquots (1x10e6 cells) were initially incubated with LIVE/DEAD Fixable Near IR Dead fluorescent viability dye (ThermoFisher Scientific, USA) for 30 min, then rinsed and pre-blocked with 5% FcR binding reagent (TruStain FcX, Biolegend), prior to 30 min staining with a panel of monoclonal antibodies [CD3ϵ FITC (1:250, clone UCHT1 gamma, produced at the Department of Immunology and Biotechnology, University of Pecs), CD4 PE-Cy7 (1:200, clone SK3, eBiosciences), CD8a PerCP-Cy5.5 (1:200, clone RPA-T8, eBiosciences), TCRVα7.2 PE (1:100, clone 3C10, BioLegend), TCRγδ PE-Cy7 (1:100, clone B1, BioLegend), TCRVδ1 APC (1:100, clone TS8.2, eBiosciences), TCRVδ2 PerCP/CY5.5 (1:200, clone B6, BioLegend)] and MR1-5-OP-RU [5-(2-oxopropylideneamino)-6-D-ribitylaminouracil] conjugated tetramers [1:100, NIH Tetramer Core Facility (38)]. Five-parameter gating strategy encompassing CD3, CD4, CD8, TCRVα7.2 and MR1-5-OP-RU tetramer surface expression was used for flow cytometry evaluation of freshly collected MR1-restricted T cells.

**Figure 1:**
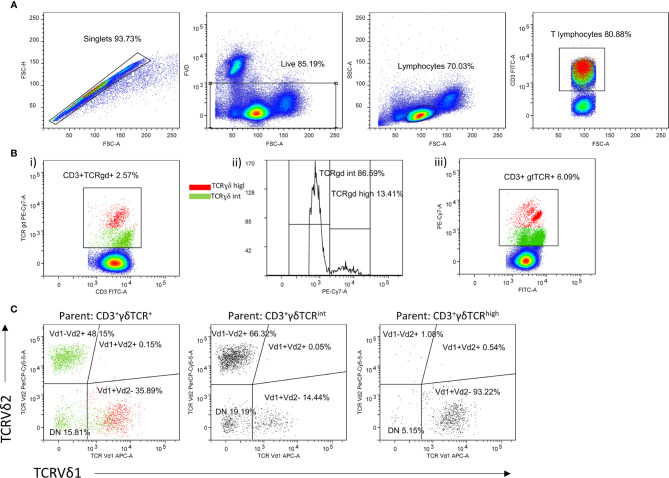
Gating strategy of peripheral γδ T cells. **(A)** Representative dot plots illustrate gating strategy for identification of live, CD3^+^ T cells, **(B)** including gating of CD3^+^γδTCR^int^ and CD3^+^γδTCR^high^ and **(C)** Vδ1^+^, Vδ2^+^ and Vδ1^-^δ2^-^ T cell subsets analyzed within each CD3^+^γδTCR^int^ and CD3^+^γδTCR^high^ T cell panel. The Vδ1^+^δ2^+^ T cell events were not further evaluated.

Initial gating strategy for peripheral γδ T cell frequency analysis included evaluation of CD3 and γδTCR cell surface expression (**Figures 1A, B**). Thereafter, we assessed γδ cell lineages in more detail, by considering TCRδ chain usage in a subset of cryopreserved PBMC samples (**Figure 1C**). For this purpose, a cross-validation was performed by using a panel of CD3, γδTCR, TCRVδ1 and TCRVδ2 antibodies to show that relative cell populations, as measured by FACS, were equivalent for paired, fresh and cryopreserved samples (**Supplementary Figure 1**). The results demonstrated that our fresh and thawed PBMCs were comparable, showing consistent cellular proportions were recovered with different sample preparation methods. Only data that passed viability control metrics (>70%) from the FACS instruments were included (n=34).

Compensation parameters were set according to the single stained samples, while fluorescence-minus-one (FMO) and isotype controls were used for gate adjustments (**Supplementary Figure 2**). Flow cytometry data were collected on BD FACS Canto II cytometer and processed with FlowLogic v7.2.1. software (Inivai Technologies, Australia). Simultaneous 2-way cell sorting was performed on a 4-color S3e cell sorter (Bio-Rad Laboratories, SAD) and was restricted to CD3^+^γδTCR^+^ and CD3^+^TCRVα7.2^+^MR1-5-OP-RU tetramer^+^ expressing T cells. A minimum of 3,000 MR1-reactive T cells and 15,000 γδ T cells from each, freshly collected PBMC sample were sort-purified directly into miRVana™ miRNA Lysis/Binding buffer (Thermo Fisher Scientific) and used immediately for RNA extraction according to the manufacturer’s instructions.

### cDNA Synthesis and RT-qPCR Experiments

cDNA synthesis was accomplished with the PrimeScript™ RT Reagent kit (Takara Bio, USA) using 100 ng of total RNA input in a 40 μl final mastermix reaction, as previously described (39) Quantity and purity of RNA samples were initially verified by OD_260_/OD_280_ ratio > 1.8. using IMPLEN NanoPhotometer P-Class P-330 (IMPLEN GmbH, Germany). Obtained cDNA samples were diluted 6-fold and used as a template for subsequent quantitative real-time PCR analysis of eight selected targets (PLZF/*ZBTB16, RUNX3, RORC, TBX21, EOMES, IL-18R, CCR6, CCR10*) and two reference genes (*ACTB* and *TBP*) using QuantStudio 5 instrument (Thermo Fisher Scientific, USA). All qPCR experiments were performed in triplicate 15 μl reactions containing 7.5 μl of TaqMan Universal PCR Master Mix II and 0.75 µl of pre-designed individual TaqMan gene expression assay (Applied Biosystems, USA). The cycling conditions were set according to the manufacturer’s guidelines and the list of TaqMan expression assays and amplicon sizes is given in **Supplementary Table 1**.

Ct values were determined with the use of QuantStudio Design & Analysis Software v 1.5.1. qPCR precision (R^2^ range 0.990–0.999) and amplification efficiency (80.4–99.2%) of all investigated targets were validated by analysis of 5-point fourfold serial dilutions of arbitrary standards that were run in parallel to samples during each experiment. Intra-assay variability was less than 1.57% and less than 2.93% in-between different qPCR experiments. Expression levels of investigated transcripts were normalized relative to the *ACTB* reference gene, validated *via* NormFinder algorithm as the best endogenous control for both MR1-reactive (M=0.359) and γδ T cell (M=0.515) sample cohort. Fold difference in mRNA expression was finally calculated according to the efficiency corrected model of 2^-ΔΔCt^ method as described by Pfaffl 2001 (40).

### Luminex Analysis of Cytokine and Chemokine Serum Levels

Peripheral blood samples for serum analysis were collected in anticoagulant-free vacutainers and centrifuged for 10 min at 1,000 g to obtain, aliquot and store (−80°C) serum samples until use. The Human Custom Procarta Plex 5-plex kit (eBioscience, Affymetrix) and the Luminex 200 platform were used for the multiplex quantitative analysis of IL-17A/F, IL-18, IL-23, CCL20, and CCL27 serum levels according to instructions in the manufacturer’s leaflet. Briefly, all samples were tested in duplicate reactions comprising 50 μl of prewashed Antibody Magnetic Bead Mixture, 25 μl of Universal Assay Buffer and 25 μl of serum. Following 2 h incubation at 500 rpm, antibody captured analytes were mixed with 25 μl of biotinylated Detection Antibody Mixture, and the 96-well plate was incubated for 30 min with constant shaking at 500 rpm. Streptavidin conjugated phycoerythrin (50 μl) was added in the next step, mixed with 120 μl of Reading buffer and incubated for 5 min at 500 rpm before reading the plate in the Luminex instrument.

The seven-point fourfold serial dilutions of absolute standards were run in parallel to the samples, and the linear regression coefficient (R2) determined for all studied analytes varied between 0.983-0.996. The upper (ULOQ) and lower limits of quantitation (LLOQ) in the 7-point serial dilution of standards were as follows: IL-17A/F (LLOQ–ULOQ; 7.25–29,700 pg/mL), IL-18 (14–14,750 pg/mL), IL-23 (15–60,900 pg/mL), CCL20 (6.49–6,650 pg/mL) and CCL27 (2.47–10,100 pg/mL). The levels of tested analytes in serum samples were determined using the 5P logistic fit algorithm of the ProcartaPlex Analyst software v 1.0. (eBioscience, Affymetrix).

### Statistical Analysis

Normality of distributions was assessed by the Shapiro-Wilk test and the homogeneity of variances by Levene’s test. Subsequently, a nonparametric approach was adopted. Continuous data are presented as median with interquartile range (IQR). Before analysis, serum CMV IgG levels were winsorized at the upper level of detection range. The Mann-Whitney U-test was used for independent group comparisons, and the Fisher’s exact test was applied to contingency tables. Wilcoxon’ signed-rank test for difference in medians was applied to paired samples. Pairwise correlations were assessed by Spearman’s rank test, whereas Lin’s concordance correlation coefficient was used for measuring agreement on a continuous scale (41). For transcriptomic data, dimensionality reduction was performed by principal component analysis (PCA) using log_2_-transformed fold-change values. Two-tailed P<0.05 was considered significant. No adjustment for multiple testing was applied, stressing the exploratory (hypotheses generating) nature of inferential statistics. If not otherwise stated, statistical analyses were performed with NCSS2007 (v07.1.20, NCSS LLC, Kaysville, Utah, USA). For PCA and graphical rendering, R software v3.6.0 (www.r-project.org) was used in RStudio v 1.2.5001 environment (RStudio Inc., Boston, MA, USA), together with ComplexHeatmap, DescTools, factoextra, FactoMineR, ggjoy, ggplot2, ggpubr, Hmisc, missMDA, pheatmap, randomcoloR, RColorBrewer, and tidyr packages.

## Results

### Demographic and Biochemical Data

Subjects’ characteristics are outlined in **Table 1**. Sex composition (Fisher’s exact P=0.75), age (P=0.922) and body mass index (P=0.356) were similar in both patients and healthy controls. Both groups had comparable CBC counts, and CRP levels [PV vs. controls: 2 (0.7–3.2) vs. 0.8 (0.5–1.8) mg/l, P=0.06]. No significant differences in CMV IgG seropositivity (13/4 vs. 17/3, pos./neg., PV vs. controls, Fisher’s exact P=0.68), serum CMV IgG levels [median (IQR): 145 (29–185) vs. 136 (72–184) AU/mL, PV vs. controls, P=0.866], and CMV IgM seropositivity (1/16 vs 0/18, pos./neg., PV vs. controls, Fisher’s P=0.485) were noticed either. Age (P=0.233), CRP (P=0.75), and CMV IgG levels did not differ significantly between men and women (P=0.726) who had serologic data available. Males, however, had higher BMI values [25.5 (23.2–27.8) vs. 21 (19.7–26.8) kg/m^2^, P=0.04]. Within the subset of donors for whom a paired, cryopreserved PBMC sample was available [18 controls (4 females), 16 PV (2 females)], higher CRP levels were noted in PV [1.9 (0.6–2.8) vs. 0.6 (0.3–1) mg/l, P=0.021], with no difference in sex composition (P=0.608) (P=0.66, Fisher’s exact test), age (P=0.628), CMV IgG titre (P=0.608), and BMI (P=0.138) between the case-control groups. All participants were negative for Mycobacterium tuberculosis infection, anti-HCV and HBsAg, while anti-HBs antibodies were detected in 43% (9/21) of patients and 55% (12/22) of controls, most likely reflecting previous immunization *via* Croatian anti-HBV compulsory vaccination program. At the time of inclusion, all patients were therapeutically naïve, with varying, but mostly mild disease severity scores ranging between 2.1–18.1 [7.7 (5.5–12.5), PASI] and 0–20 [3.0 (1–6.5), DLQI]. No association was observed between PASI or DLQI score and anti-CMV IgG or anti-HBs antibody titre.

**Table 1:** Demographic, clinical, and biochemical characteristics of patients living with psoriasis (PV) and healthy controls.

Group	PV	Controls	P
**N (M/F ratio)**	21 (14/7)	22 (16/6)	0.75**
**Age (years)**	33 (27–39)	32 (28–40)	0.922*
**BMI (kg/m^2^)**	25.2 (21.3–29.1)	24.7 (20.7–27.1)	0.356*
**hsCRP (mg/L)**	2 (0.7–3.2)	0.8 (0.5–1.8)	0.06*
**PASI**	7.7 (5.5–12.5)	–	–
**DLQI**	3.0 (1.0–6.5)	–	–
**Anti-CMV IgG (pos/neg)**	13/4	17/3	0.68**
**Anti-CMV IgG (AU/mL)**	145 (29–185)	136 (72–184)	0.866*
**Anti-CMV IgM (pos/neg)**	1/16	0/18	0.485**
**Anti-HBs IgG (mIU/mL)**	28 (0–153)	201 (16–725)	0.095*

* Mann-Whitney U-test.

** Fisher’s exact test.

Data are presented as medians with interquartile range (IQR). N, number of participants; BMI, body mass index; hsCRP, high-sensitivity C reactive protein; PASI, Psoriasis Area and Severity Index; DLQI, Dermatological Life Quality Index; CMV, Cytomegalovirus; HB, hepatitis B; IgG/M, Immunoglobulin G/M.

### Cytokine and Chemokine Serum Levels in PV Patients and Healthy Controls

The IL-17A/F, IL-23, and CCL20 serum levels were below lower limits of quantitation in all tested samples. Serum values of two detectable analytes, i.e. CCL27, a CCR10 ligand which promotes CD162(CLA)^+^ T cell trafficking to epithelial sites, and IL-18, varied between 66.78–618.04 pg/ml, and 3.22–23.57 pg/ml across the whole sample collection, respectively. In case-control comparisons, no significant difference in either CCL27 [PV vs. CTRL: 352.7 (267.4–496.6) vs. 343.5 (203.6–487.7) pg/ml, P=0.704] or IL-18 [PV vs. CTRL: 9.1 (8.1–13.1) vs. 9.2 (8.3–10.7) pg/ml, P=0.64] serum levels was observed. Similarly, no significant relationship was observed between either CCL27 or IL-18 serum levels, disease severity, CMV IgG titer, age or sex (data not shown).

### Distinct CD3^+^γδTCR^+^ and TCRδ Subsets Are Differentially Altered by BMI, CMV Status, and Serum Cytokine Content

**Figure 2:**
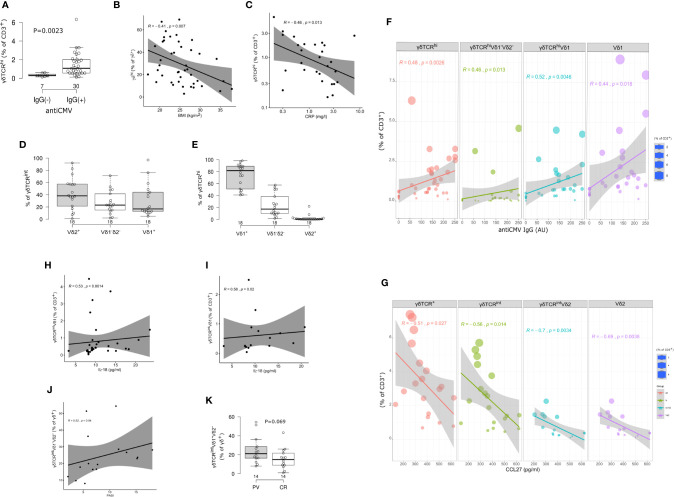
Circulating γδ T cell compartment of adults is skewed by CMV status, body mass index (BMI), inflammatory burden, and serum cytokine content. **(A)** Boxplot showing circulating γδTCR^high^ T cells frequency in annotated groups, according to the CMV status (two-tailed Mann-Whitney test, pooled sample, PV cases + controls). Horizontal lines represent median with interquartile range. **(B)** γδTCR^high^ blood T cells frequency is inversely related to body mass index (BMI, pooled sample). R denotes Spearman’s correlation coefficient. The black line represents a linear model fit where the shaded region indicates 95% confidence interval. **(C)** The relationship between circulating γδTCR^high^ T cell frequency and C-reactive protein (CRP) at the time of blood sampling (log-log scale, pooled sample, cases+controls). **(D, E)** TCRδ chain usage in blood γδ T cells, healthy controls. Horizontal lines represent median with interquartile range. **(F, G)** Frequency and phenotype of circulating γδ T cells co-vary with anti-CMV IgG levels (pooled sample, PV+controls) and serum CCL27 levels (PV cases). The size of the dot reflects the percentage of cells expressing the markers, while the color encodes cell type identity within the group. Linear fit model, the shaded region indicates 95% confidence interval. **(H, I)** Correlation between Vδ1^+^ γδTCR^int^ T cell frequency (peripheral blood) and serum IL-18 levels. h: pooled sample, i: PV cases. **(J)** Correlation between Vδ1^-^Vδ2^-^ subset of γδTCR^int^ cells and disease severity, measured by PASI score, at the time of blood sampling. **(K)** Boxplot showing circulating Vδ1^-^Vδ2^-^ γδTCR^int^ T cells frequency in annotated groups (*males* PV, CR=controls, two-tailed Mann-Whitney test). CMV status, BMI, and CRP were considered a shared covariate, common to each (case and control) study arm.

In line with previous reports (22), two circulating γδ T cell populations could be distinguished in most individuals, giving rise to CD3^+^γδTCR^int^ cells, and a smaller, CD3^+^γδTCR^high^ fraction (**Figures 1B I, II**, **Table 2**). For some donors, however, more-diffuse staining patterns were observed (**Figure 1B III**). Overall, CMV seropositive (**Figure 2A**) and lean subjects (**Figure 2B**) shared an expansion of CD3^+^γδTCR^high^ cells at systemic level, supporting a prominent position of γδ T cell communities in CMV defence (42) and adipose tissue biology (23). By contrast, the counts of CD3^+^γδTCR^high^ cells declined with higher acute-phase inflammatory burden (**Figure 2C**). With regard to TCRδ chain usage, γδTCR^int^ cells were mostly Vδ2^+^, outnumbering Vδ1^+^ and Vδ1^-^Vδ2^-^ subsets (**Figure 2D**). Conversely, Vδ1^+^ cells dominated the CD3^+^γδTCR^high^ subset (**Figure 2E**), more so in CMV-experienced than CMV-naive individuals (**Figure 2F**), with only a paucity of γδTCR^high^ cells expressing Vδ2 chain. For the Vδ1^-^Vδ2^-^ subset of γδTCR^high^ cells, a weak enrichment in highly antiCMVIgG-positive subjects (**Figure 2F**) confirmed that the human CMV response is not restricted to Vδ1 population (43). Overall, CMV status and BMI emerged as major covariates underlying TCRδ profiles in circulating CD3^+^γδTCR^high^ cells.

**Table 2:** Peripheral blood frequencies of CD3^+^γδTCR^high,^ and CD3^+^γδTCR^int^ T cells (freshly isolated PBMC) in healthy controls (n=22) and PV patients (n=21).

CD3^+^γδTCR^+^	Controls	PV	Mann-Whitney P
**CD3^+^γδTCR^high^**			
% CD3^+^	0.78 (0.32–1.97)	0.76 (0.42–1.74)	0.761
% CD3^+^γδTCR^+^	23 (12.4–46.4)	28.1 (15.5–46.2)	0.671
**CD3^+^γδTCR^int^**			
% CD3^+^	2.53 (1.53–5.1)	2.27 (0.98–4.68)	0.536
% CD3^+^γδTCR^+^	77 (53.6–87.6)	71.9 (53.8–84.3)	0.671

Data are presented as medians with interquartile range (IQR).

Serum cytokine content further modified these proportions, principally affecting γδTCR^int^ compartment and its TCRδ composition. Specifically, the peripheral abundance of total CD3^+^γδTCR^+^ and CD3^+^γδTCR^int^ cells diminished with increasing serum CCL27, largely in response to declining Vδ2^+^ cell numbers (**Figure 2G**), and this association appeared restricted to subjects with PV (**Supplementary Figure 3**). In a similar vein, the frequency of Vδ1^+^ γδTCR^int^ T cells positively correlated with IL-18 serum levels (**Figures 2H, I**, **Supplementary Figure 4**). Taken together, these results reveal potentially distinct patterns of rewiring in various TCRδ subsets of γδTCR^int^ cells, conditional on PV status.

### PV Is Associated With Multiple γδ Blood Cell Populations

Having established several biological sources of confounding which could obscure the true effect under realistic conditions, we finally tested for case-control differences using sex, age, BMI and CMV-matched individuals. This showed that numerical profiles of circulating Vδ2^+^ γδTCR^high^ and Vδ1^-^δ2^-^ γδTCR^int^ T cells are coordinated in PV [Spearman’s ρ(PV)=0.52, P=0.04, n=16; ρ(PV+controls)=0.4, P=0.02, n=34]: Vδ2^+^ γδTCR^high^ compartment was significantly expanded in γδ T cells of male psoriatic patients compared to healthy male (**Table 3**), but not across the whole sample (**Supplementary Table 2** and **Supplementary Table 3**; the number of female participants was too low for a meaningful comparison). As judged by PASI, Vδ1^-^δ2^-^ γδTCR^int^ T cell numbers increased with the severity of disease (**Figure 2J**), leading to a marginally higher proportion of Vδ1^-^δ2^-^ γδTCR^int^ T cells in affected male donors (**Figure 2K**). Of note, age (P=1), BMI (P=0.74), and serum CMV IgG levels did not differ significantly between male controls and male patients [141 (76–217) vs. 123 (0–153) AU/ml, 14 vs. 10 individuals, P=0.177]. There were no significant cell proportion differences for any other γδ subpopulation.

**Table 3:** Peripheral blood proportions of γδTCR^high,^ γδTCR^int^ T cells (cryopreserved PBMC, % CD3^+^γδTCR^+^), and their respective TCRδ subsets (median, interquartile range) in healthy male controls (n=14) and male PV patients (n=14).

γδTCR^+^	Controls (%)	PV (%)	Mann-Whitney P
**γδTCR^high^**			
Vδ1^+^Vδ2^-^	24.1 (9.4–34.5)	26.6 (10.5–29.4)	0.872
Vδ1^-^Vδ2^+^	0.08 (0–0.23)	0.25 (0.09–0.68)	0.04
Vδ1^-^Vδ2^-^	3.5 (1.5–7.3)	3.4 (1.6–5.6)	1
**γδTCR^int^**			
Vδ1^+^Vδ2^-^	13.2 (8.9–34.7)	16.8 (5.9–32.8)	0.836
Vδ1^−^Vδ2^+^	29 (14.5–54.3)	18.5 (9–35.6)	0.346
Vδ1^−^Vδ2^−^	15 (8.5–22.4)	21.1 (15.3–30.5)	0.069

Data are presented as medians with interquartile range (IQR).

### γδ Transcriptome Is Affected by Changes in Cell Type Composition and PV

**Figure 3:**
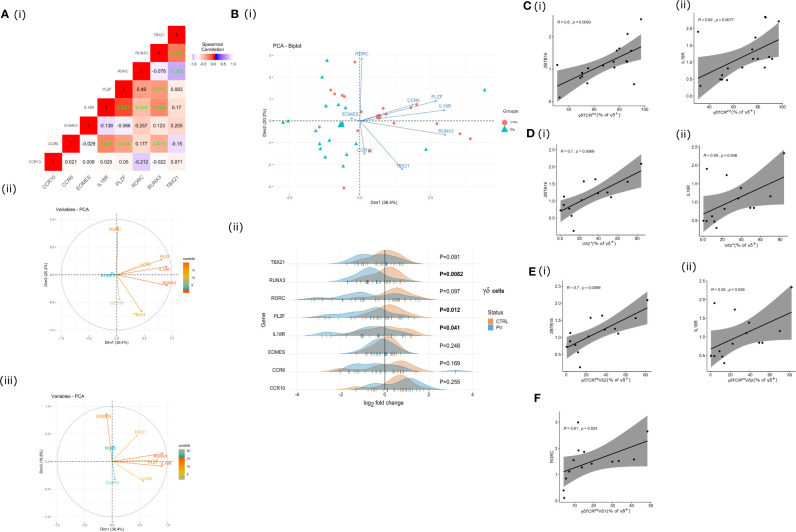
γδ T cell transcriptome is affected by changes in cell type composition and PV. **A** (I) Correlation heatmap depicting bulk expression of transcription factors/chemokine receptors in circulating γδ T cells (Spearman’s correlation coefficient, healthy controls). Statistically significant correlations (two-tailed P<0.05) are denoted in green. **A** (II), **A** (III), **B** (I) The PCA biplots **A** (II), **A** (II) and score plot **B** (I), transcriptomic data, γδ blood T cells (pooled sample). Positively correlated transcripts point to the same side of the plot. The score plot shows the degree of discrimination that was achievable across the groups. Each dot represents an individual: healthy controls (red) and PV cases (green). **B** (II) Ridgeline plots showing gene expression values of depicted genes in two clusters (healthy controls, PV cases). For differentially expressed transcripts (P<0.05, two-tailed Mann-Whitney test), P-values are depicted in bold. Each “|” point shape represents an individual. **C** (I), **C** (II), **D** (I), **D** (II), **E** (I), **E** (II), **F** Heterogeneous bulk mRNA expression in flow-sorted γδ T cells, healthy controls. Scatterplots showing co-variations between indicated gene expression (ZBTB16, IL18R, RORC; fold change) and γδ T cell composition. *R* denotes Spearman’s correlation coefficient. The black line represents a linear model fit where the shaded region indicates 95% confidence interval.

To better characterize the biological features of γδ blood T cells in psoriasis, we next assessed the transcriptional profile of flow-sorted γδ population by targeting several genes essential to development of innateness (*ZBTB16, RUNX3, IL18R*), type 17 response (*CCR6, RORC*), Th1/cytotoxic polarization (*RUNX3, TBX21, EOMES*), and tissue homing (*CCR6*, *CCR10*). This revealed that *IL18R* and *ZBTB16* followed a similar co-expression pattern (**Figures 3A I, II, III**), and were coordinately depleted in PV vs. controls (**Figure 3B I**), together with *RUNX3* (**Figure 3B II**). No significant differences were observed for any other tested gene. As anticipated, *ZBTB16* and *IL18R* expression levels strongly co-varied with cell composition at baseline, paralleling the proportion of CD3^+^γδTCR^int^ (**Figures 3C I, II**), Vδ2^+^ (**Figures 3D I, II**), and Vδ2^+^ γδTCR^int^ cells (**Figures 3E I, II**) in γδ T cell mixture, but no evidence of exquisite restriction to a single compartment was observed. *RUNX3* expression, which reportedly promotes the maturation of DN TCRγδ^+^ thymocytes (44, 45), was broader, and apparently not constrained to any major cell subset. In healthy controls, bulk *RORC* expression co-ordinately increased with the relative size of the Vδ1^+^ γδTCR^int^ subset (**Figure 3F**), suggesting that these TFs might operate in different cellular compartments. Altogether, these results indicate that PV might promote numerical and transcriptomic reorganization of the γδ cytome early in disease course, at least in type I PV.

### CD4^+^ and DP MAIT Cell Compartments Are Reduced in Peripheral Blood of Male Psoriatic Patients

**Figure 4:**
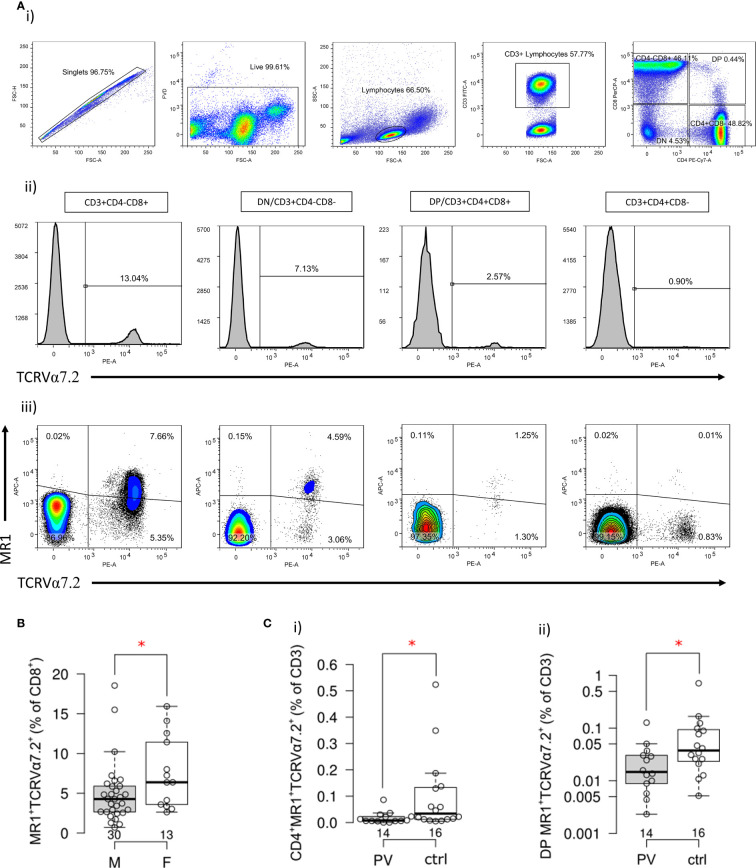
Frequency and phenotype of MR1-restricted MAIT cells in PV patients and healthy controls. **(A)** Representative dot plots illustrate gating strategy of MR1-5-OP-RU tetramer positive (MR1-tet^+^)TCRVα7.2^+^, MR1-tet^+^TCRVα7.2^-^, MR1-tet^-^TCRVα7.2^+^ and MR1-tet^-^TCRVα7.2^-^ T cells within CD4^+^, CD8^+^, CD4^-^CD8^-^ (DN) and CD4^+^CD8^+^ (DP) peripheral T cell compartment analyzed by multiparameter flow cytometry. **(B)** Male examinees have significantly lower frequency of circulating CD8^+^MR1-tet^+^TCRVα7.2^+^ cells then age-matched women, and c) compared to healthy male controls, a significantly smaller fraction of (I) CD4^+^ and (II) DP MR1-tet^+^TCRVα7.2^+^ MAIT cells within the circulating CD3^+^ T cell pool [Mann-Whitney test **(B, C)**, P<0.05 is considered significant and illustrated by red asterisk; horizontal lines represent median with interquartile range (IQR), note that Y-axis **(C** II) is log scale].

To better delineate differences in MR1-restricted cell subpopulations between psoriatic patients and healthy controls (**Table 4**), peripheral CD4^+^, CD8^+^, DP and DN T cells were profiled by MR1-5-OP-RU tetramer and TCRVα7.2 surface staining. Subsequently, four different T cell subpopulations were identified and further analyzed within each (CD4^+^, CD8^+^, DP or DN) compartment (**Figures 4A I, II**): MR1-5-OP-RU tetramer^+^ TCRVα7.2^+^(MAIT) cells, MR1-tet^+^TCRVα7.2^-^ (atypical MR1-reactive T cells) (46), MR1-tet^-^TCRVα7.2^+^, and MR1-tet^-^TCRVα7.2^-^ cells. CD3^+^MR1-5-OP-RU tet^+^ cells were retained for further analysis.

**Table 4:** Peripheral blood frequencies of canonical and atypical MR1-restricted T cells in healthy controls (n=22) and PV patients (n=21).

CD3^+^MR1-tet^+^TCRVα7.2^+^	Controls (%)	PV (%)	Mann-Whitney P
CD4^-^CD8^-^	0.13 (0.08–0.38)	0.14 (0.04–0.28)	0.636
CD4^-^CD8^+^	2.1 (1.2–3.6)	1.6 (0.9–2.5)	0.22
CD4^+^CD8^+^	0.04 (0.02–0.08)	0.03 (0.009–0.05)	0.106
CD4^+^CD8^-^	0.02 (0.006–0.13)	0.01 (0.006–0.026)	0.17
All	2.3 (1.3–4.2)	1.8 (1.1–3.1)	0.185
**CD3^+^MR1-tet^+^TCRVα7.2^-^**			
CD4^-^CD8^-^	0.005 (0.003–0.011)	0.003 (0.0007–0.0084)	0.091
CD4^-^CD8^+^	0.026 (0.015–0.059)	0.025 (0.013–0.034)	0.504
CD4^+^CD8^+^	0.0014 (0.0006–0.0028)	0.0022 (0.001–0.0042)	0.324
CD4^+^CD8^-^	0.023 (0.01–0.038)	0.024 (0.014–0.034)	0.706
All	0.06 (0.034–0.12)	0.06 (0.04–0.084)	0.504

Data are presented as medians with interquartile range (IQR).

As expected, circulating MR1-tet^+^ TCRVα7.2^+^T cells of healthy examinees were most often CD8^+^ and DN, whereas only a minority was DP or CD4^+^ (**Figure 4A III**, **Table 4**). Male subjects had fewer circulating MR1-tet^+^ TCRVα7.2^+^cells within their CD8^+^fraction [pooled samples, PV+controls, F vs. M: 6.4 (3.5–12) vs. 4.3 (2.6–6) %, P=0.049, **Figure 4B**], which aligned with previous reports on higher MAIT cell proportions in women vs. age-matched men (47). In case-control comparisons, CD4^+^MR1-tet^+^TCRVα7.2^+^ [PV vs controls: 0.007 (0.005–0.025) % vs. 0.034 (0.012–0.135) %, P=0.017, **Figure 4C I**] and DP MR1-tet^+^TCRVα7.2^+^ [PV vs. controls: 0.015 (0.008–0.032) % vs. 0.038 (0.022–0.096) %, P=0.032, **Figure 4C II**] cells occupied a significantly smaller fraction of the CD3^+^T cell pool in male psoriatic patients when compared to healthy male controls. In addition, an inverse relationship was observed between the relative proportions of DP MR1^+^TCRVα7.2^+^ and CD3^+^γVδ1^-^δ2^-^TCR^int^ cells [ρ(PV+controls)=−0.39, P=0.024, n=34, cases+controls], suggesting concurrent variability in two differentially represented, PV-associated cell subsets. No difference was observed for any other remaining target cell population in full-sample and sex-stratified (males-only) case-control comparisons.

### Atypical MR1-Reactive T Cells Are Not Numerically Altered, but Inversely Correlate With IL-18 Serum Levels in PV Patients

**Figure 5:**
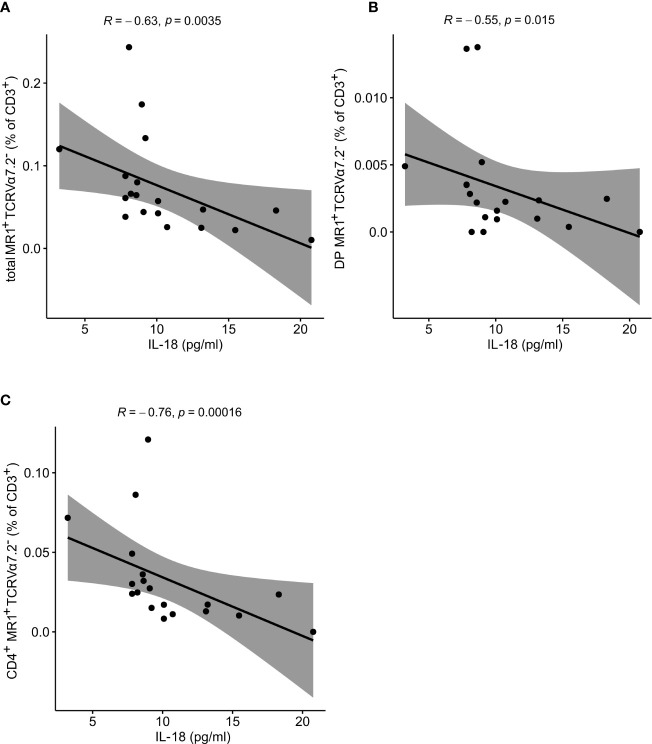
Atypical MR1-restricted Vα7.2^-^ T cells correlate with cytokine serum levels of PV patients. The peripheral frequency of atypical **(A)** CD3^+^, **(B)** DP and **(C)** CD4^+^TCRVα7.2^-^ T cell compartments negatively correlate with IL-18 serum levels of PV patients, respectively (R denotes Spearman’s correlation coefficient. The black line represents a linear model fit where the shaded region indicates 95% confidence interval).

In the next step, the atypical MR1-reactive, but TCRVα7.2-negative peripheral T cell pool was enumerated as well. In line with previous reports on human atypical MR1-restricted αβ T cell compartment [40], majority of circulating MR1-tet^+^TCRVα7.2^-^ T cells of healthy controls (**Table 4**) were either CD8^+^ or CD4^+^, whereas minority was DN or DP. In our PV dataset, the total size of the atypical CD3^+^MR1-restricted, TCRVα7.2^-^ compartment was inversely related to serum IL-18 levels (**Figure 5A**), whereby peripheral DP (**Figure 5B**) and CD4^+^ (**Figure 5C**) MR1-tet^+^TCRVα7.2^-^ T cell subsets were largely responsible for the observed effect. No association was observed with the case-control status, CMV seropositivity, sex and age (data not shown) for any atypical MR1-restricted T cell class.

### *RORC*, *CCRC*6, and *EOMES* Transcripts Are Differentially Expressed in Circulating MR1-tet^+^ TCRVα7.2^+^T Cells of PV Patients

**Figure 6:**
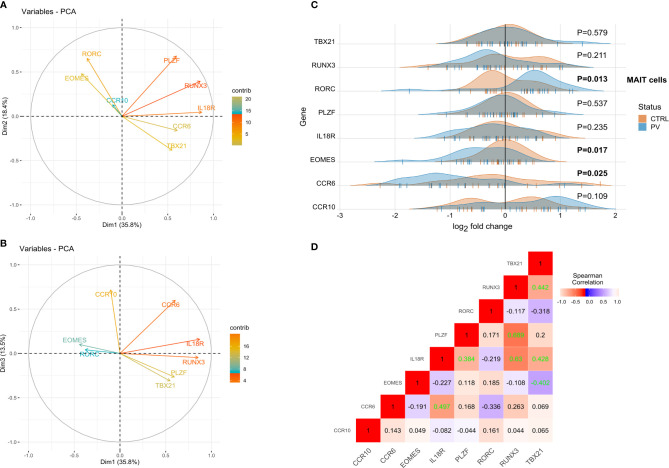
*RORC*, *CCRC*6 and *EOMES* transcripts are differentially expressed in circulating MR1-tet^+^ TCRVα7.2^+^T cells of PV patients. PCA biplots **(A, B)**, transcriptomic data, MAIT blood cells (pooled sample). Positively correlated transcripts point to the same side of the plot. Negatively correlated variables point to opposite sides of the graph. **(C)** Ridgeline plots showing gene expression values of depicted genes in two clusters (healthy controls, PV cases). For differentially expressed transcripts (P<0.05, two-tailed Mann-Whitney test), P-values are depicted in bold. Each “|” point shape represents an individual. **(D)** Correlation heatmap depicting bulk expression of transcription factors/chemokine receptors in circulating MAIT cells (Spearman’s correlation coefficient, healthy controls). Statistically significant correlations (two-tailed P<0.05) are denoted in green.

The transcriptional profile of purified blood MR1-tet^+^ TCRVα7.2^+^T cells was assayed next. In view of developmental similarities between MAIT and γδ T cells (48), we probed the same selection of target genes *ex vivo via* RT-qPCR (**Figures 6A, B**). As a result, an overlapping correlation pattern, which was significantly stronger for markers of innateness as compared to the other genes, emerged by comparing MAIT and γδ T cells. Among the differentially expressed genes (**Figures 6C, D**), transcripts whose products are involved in MAIT17 response (*RORC*, *CCR6*), tissue residency (*CCR6*), memory-like differentiation, and cytotoxicity (*EOMES*) were observed. While *RORC* expression was significantly up-regulated in conventional MAIT cells of PV patients, *EOMES* was down-regulated compared to controls, evoking differential, mutually exclusive requirements for lineage decisions in Tc17 and metabolite-specific T cells (48, 49). Interestingly, *CCR6*, which is involved in thymic egress and guides tissue localization of other innate-like T cells, was down-regulated in PV, but the significance of this finding for MR1-restricted T cells and PV remains to be addressed. No significant associations were observed for age, sex, BMI, anti-CMV IgG, serum cytokine levels, disease severity, and CD4/CD8 cell composition (data not shown; note, however, that this kind of deconvolution is inherently inefficient for rare cell populations, such as CD4^+^MAIT cells). Overall, these data suggest that the transcriptomic response of circulating innate-like T cells evolves parallely and early in disease course of PV, in a manner that differs fundamentally between γδ and MAIT cell populations.

## Discussion

Despite numerous animal studies, the composition of innate-like T cells, and their contribution to human PV remain ambiguous. Here, we dissect the diversity of γδ and MR1-restricted blood T cells in untreated, mildly affected PV patients, and show that the largest effect on their compositional profile was exerted by CMV exposure, BMI status, and sex, respectively, which largely, but not completely overpowered the disease effect. As a result, we propose that multiple, circulating innate-like T cell subsets undergo a range of distinct, previously unrecognized compositional alterations in PV, by identifying novel subcommunities with significant numerical changes in male PV patients. Of these, a minor subset of canonical CD4^+^ (SP and DP) MR1-tet^+^TCRVα7.2^+^ T cells was the most significantly underrepresented community during type I disease, which was absent or low in affected male individuals, irrespectively of PASI/DLQI measures. Likewise, the proportion of circulating, very sparsely represented Vδ2^+^ γδTCR^high^ and Vδ1^-^δ2^-^ γδTCR^int^ was increased in male patients, the latter mirroring disease activity, while no association was seen for the entire, sex-mixed PV cohort. No evidence of case-control association was observed for canonical CD4^-^CD8^+^ and CD4^-^CD8^-^ (DN) MAIT cells, or for any other subset of atypical MR1-restricted TCRVα7.2^-^ blood T cells, at least within the spectrum of mildly to moderately affected young adults. In line, no alterations in measured serum chemokine levels (IL-17A/F, IL-23, IL-18, CCL20, and CCL27) were observed either, confirming a weak relationship between serum cytokines and skin changes in all but most severely affected individuals (50–53). Instead, a relative depletion of circulating Vδ2^+^ γδTCR^int^, and atypical CD4^+^CD8^+^ (DP) and CD4^+^CD8^-^ MR1-restricted T cells was observed in the face of increasing CCL27 and IL-18 levels in PV sera, respectively, possibly reflecting their different sensitivity to activation-induced cell death (54), or homeostatic trafficking and redistribution of two cell populations competitively best poised to respond to CCR10 and IL-18R ligation (55). CCL27, together with IL-18, is prominently expressed in keratinocytes (56–58), acting as a chemoattractant for a subset of skin-specific CCR10^+^γδ cells in mice (59) and humans (10). For murine MAIT and Treg cells, IL-18 may play a similar role in lungs and thymus, respectively (60, 61). How IL-18 regulates these migratory events is at present elusive; however, IL-18-dependent induction of the key homing chemokine receptor – CCR6 on thymic IL-18R^+^Tregs may provide some cues (61). Echoing these findings, a robust decline in human blood CCR10^+^ and CCR6^+^ Vγ9Vδ2 cells has been observed in advanced PV, mirroring their cutaneous accumulation in psoriatic lesions (10).

Together, these observations revealed that affected individuals had different analyte:cytome co-associations compared to those in healthy donors, indicating that innate T cell subsets might be coordinated differently in PV and healthy participants. A similar, significant increase in the degree of correlation has been recently demonstrated in prediabetes (62) and in cotwins (63) showing signs of early subclinical neuroinflammation, suggesting that very early disease stages may indeed be associated with changes in blood components when using multiple, orthogonal “omic” signatures. Nevertheless, the molecular mechanisms underlying these associations remain elusive, precluding a distinction between the cause and effect: bystander activation (64), microbial dysbiosis (65, 66), and confounding by unknown modifiers could all play a role. Furthermore, the generalizability of these findings to female patients remains an issue, because we could not efficiently test or control for many non-heritable and heritable modifiers (such as HLA composition) in our small cohort of women, raising the need for independent replication in a well-powered, longitudinal study. In addition, sampling variations, incomplete data sets, and batch effects may create analytic difficulties. Meanwhile, more details on potential sex-related differences in human immune responses have become available elsewhere (67).

Human MR1-restricted αβ T cells display a profound functional and compositional heterogeneity (26, 27, 46), presenting with a unique opportunity to shape immune responses. In the context of human PV (11) and psoriatic arthritis (33), MAIT cells have been evaluated in skin samples and synovial fluid, respectively, using TRAV1-2, CD161 and IL-18Rα as surrogate markers that relatively accurately estimate CD8^+^ and DN, but not CD4^+^ and DP MAIT cells. No difference in skin composition has been reported regarding CD8^+^CD161^+^TCRVα7.2^+^ T cell frequency in PV and healthy controls (11), but the exact number, as well as the actual contribution of other MR1-restricted, skin or blood T cell subsets to PV have remained unaddressed. We complement and expand these findings by reporting an inverse association of canonical (TCRVα7.2^+^) circulating MR1-tet^+^ CD4^+^CD8^-^ and CD4^+^CD8^+^, but not CD4^-^CD8^+^ and CD4^-^CD8^-^, or MR1-restricted TCRVα7.2^-^ T cells with PV, adding to a growing body of literature on immune cell aberrations in PV. These cells exhibit sex-based differences in the prevalence of TRAV1-2^+^ sets (47), and their canonical CD4^-^CD8^±^ fractions commonly decline among adult PBMC in response to various (auto)inflammatory processes (68–70), and aging (47, 71). In this context, it is significant that stimulation of MAIT cells may itself result in TCRVα7.2 downregulation, potentially affecting subsequent detection by flow cytometry (72, 73), but this has yet to be demonstrated *in vivo*. Conversely, a minor CD4^+^ and TRAV1-2^-^ cell subset predominate in neonates (74), and show differential cytokine production, TCR pairing and antigen reactivity (27), but have not been studied in the context of human pathology yet. The number of the latter in the blood, however, is invariably low (27, 46), limiting the conclusiveness of our results; thus, their functional role, and differentiation status in PV should be further investigated. Furthermore, γδ T cell lineage also contains a minor subset of MR1-reactive Vδ1/δ3 cells (75); consequently, a modified gating strategy would be necessary to disentangle these cells from their TCRβ^+^ MR1-tet^+^TCRVα7.2^-^ counterparts, highlighting the limits of our work. Accordingly, *in situ* demonstration of MR1-restricted T cells will be required to establish their translational potential.

The diversity of MAIT cell phenotype is also reflected at transcriptional level, varying according to their developmental stage, tissue localization, activation status, and CD4/CD8 census (49). Here, MAIT blood cells demonstrated several transcriptional differences in PV, surpassing their numerical variations. Within this module, RORC and CCR6 are involved in type-3 (MAIT17) ontogeny, mucosal residence, and early TCR activation of MAIT cells (48, 76). By contrast, EOMES, which marks CD8^+^ and early TCR-activated CD4^-^CD8^-^ MAIT cells (76), controls key checkpoints of cytotoxic maturation and exhaustion, suggesting a coordinated, multifaceted transcriptional reprogramming of MAIT cytome emerges early in PV. mRNA, however, is a poor proxy for protein expression, thus, the functional relevance of these findings is currently unknown. Consequently, there is a need for studying the cell types and states within the tissue, paving the way for potential multiomic, and single cell genomic efforts. Additionally, the patients with a more severe PV presentation should also be examined.

Next to MAIT cells, γδT lymphocytes are major innate IL-17 producers that richly populate dermal layers of lesional skin (8). These dermal populations are locally maintained (77), receive input from circulating precursor (15), and may disseminate to aggravate inflammation at distant sites (17). The full repertoire of human skin and blood γδ T cells, however, has yet to be determined. Recently, several distinct communities (γδTCR^int^ and γδTCR^hi^), which differ in TCRδ chain composition, IL-17 production, and transcriptional drivers (PLZF^hi^ vs PLZF^lo^GATA3^+^T-bet^lo^), have been described in human γδ blood cells (22). However, very little is known about how this heterogeneity in human γδ cells relates to PV. Building on this census, we first show that CMV exposure and BMI status reciprocally shape γδTCR^int^:γδTCR^hi^ ratio, mostly through the accumulation of predominantly Vδ2^lo^ and γδTCR^hi^ blood cells in CMV experienced, and lean subjects having low acute-phase inflammatory burden. As expected, the baseline, bulk γδ transcriptome aligned with interpersonal differences in cell composition, broadly mirroring the findings from Venken et al (22). In the next step, we demonstrated that circulating Vδ2^+^ γδTCR^high^ and Vδ1^-^δ2^-^ γδTCR^int^ T cells are relatively enriched in mildly affected, therapeutically-naïve males with type I psoriasis compared to age, CMV, BMI, and sex-matched baseline, partly in relation to disease severity. The picture that emerges is distinct and complementary to the earlier study, whereby a decline in circulating Vγ9Vδ2 T cells was observed in more severely affected, heavily pretreated and older patients (10), for whom CMV status and BMI remain unknown. From scRNA-seq data, it is also evident that Vγ9Vδ2 T cells are actually a heterogeneous population, comprising Th1-like γδ and Th17-like γδ cells (78). Consequently, we still lack the clear understanding of the earliest events in blood γδ cytome, at both the cellular and molecular levels. Beyond these compositional differences, we also uncover formerly unappreciated relationships between γδ transcriptional phenotype and PV, by showing a loss of innateness-associated transcription markers in bulk γδ blood transcriptome. Of those, PLZF, a TF central to the lineage commitment of innate-like T cells (79), has been associated with type-2 and type-3 cell fates (78), Vδ2^+^ and γδTCR^int^ sets (22), cytokine receptor activity (80), and cell survival (81). Clearly, deep, unbiased characterization of human γδ cells is necessary to better pinpoint the subsets underlying PV associations; meanwhile, these results hint that circulating γδ T set may be disproportionately altered already in mildly affected male patients. In support, the lesional and non-lesional skin in PV shows many shared features across the epithelial and immune compartments (82–84), including the increased presence of certain γδ subsets (85).

In summary, we found that mildly-to-moderately affected male patients display distinct numerical and transcriptional profiles of association between PV and certain understudied innate-like T cell subsets in peripheral blood. We also show here that identification of culprit cell subpopulations in human datasets is beset by extensive confounding from multiple sources, motivating new work to make the currently unresolved issues more tractable. Using this observation, we inform the ongoing discussion by dissecting the factors that drive the complexity of γδ, and proper MR1-restricted blood T cells, in human PV.

## Data Availability Statement

The original contributions presented in the study are included in the article/**Supplementary Material**. Further inquiries can be directed to the corresponding authors.

## Ethics Statement

The studies involving human participants were reviewed and approved by Osijek University Hospital (number: R2-9042/2018) and the Faculty of Medicine in Osijek (number: 2158-61-07-18-135). The patients/participants provided their written informed consent to participate in this study.

## Author Contributions

MŠ, MM, and ST developed the research concept. VP, MŠ, MM, and ST performed experiments and analysed data. VP, MM, MT, and IM recruited patients. MŠ performed statistics. MŠ, MM, and ST produced figures. VP, MŠ, MM, and ST interpreted data and wrote the paper. MŠ, LG-O, MP, and PB supervised the analysis, reviewed, and edited original draft. ST secured funding acquisition. All authors contributed to the article and approved the submitted version.

## Funding

This research was funded by the Intramural Research Programme of the Josip Juraj Strossmayer University (grant number VIF2018-MEFOS-8, IP-2019-8, UNIOS-ZUP-2018-22), Croatian Science Foundation (grant number UIP-2019-04-3494) and in part by Palacky University (IGA PU: LF_2020_004).

## Conflict of Interest

The authors declare that the research was conducted in the absence of any commercial or financial relationships that could be construed as a potential conflict of interest.
