# Supplementary figures and images for: Differential Skewing of Circulating MR1-Restricted and γδ T Cells in Human Psoriasis Vulgaris
Source: Front Immunol. 2020 Dec 3;11:572924. doi: 10.3389/fimmu.2020.572924 (PMC7744298; doi:10.3389/fimmu.2020.572924)

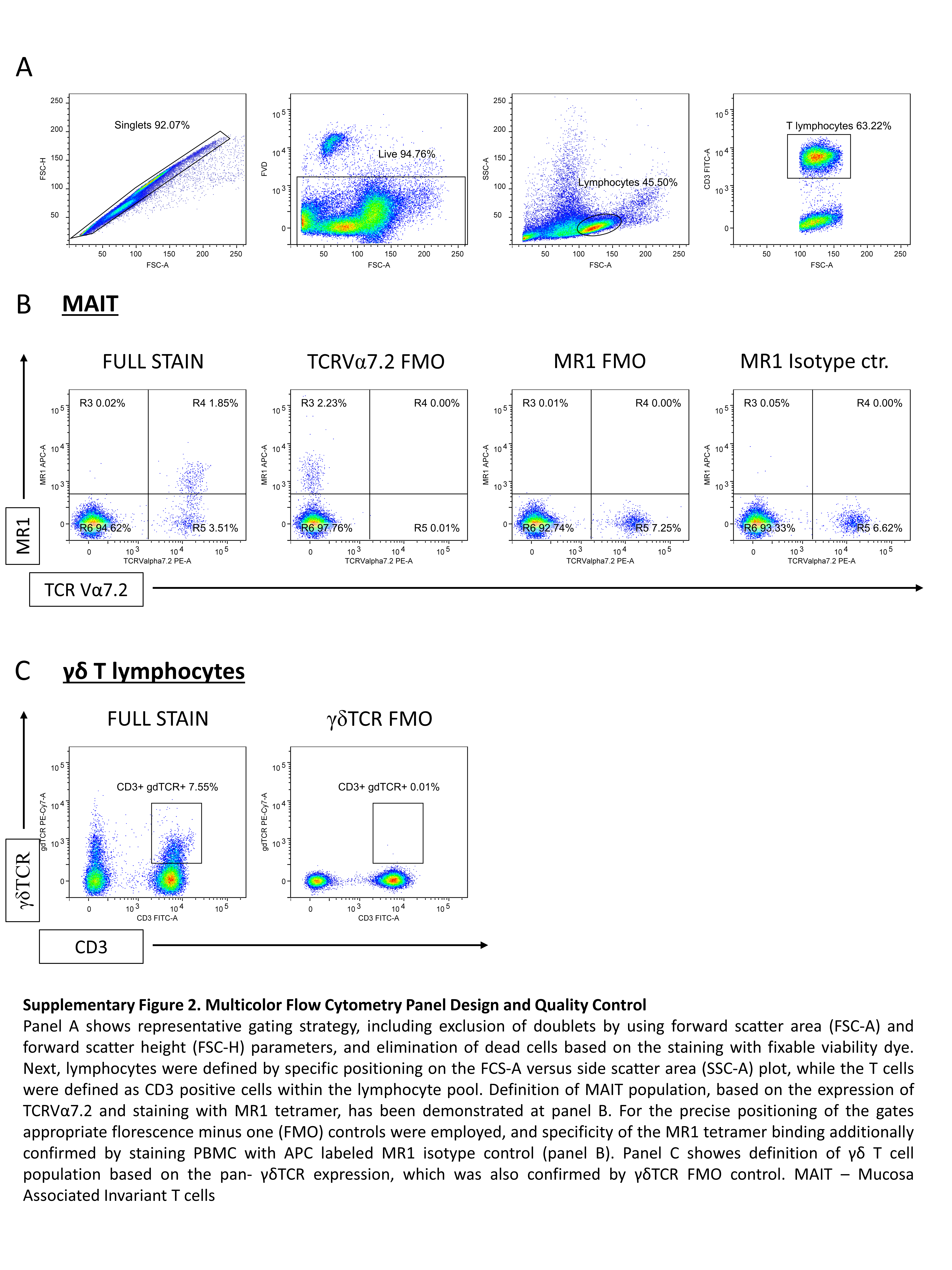

Supplement: Supplementary file 2 [file Image_2.tif]

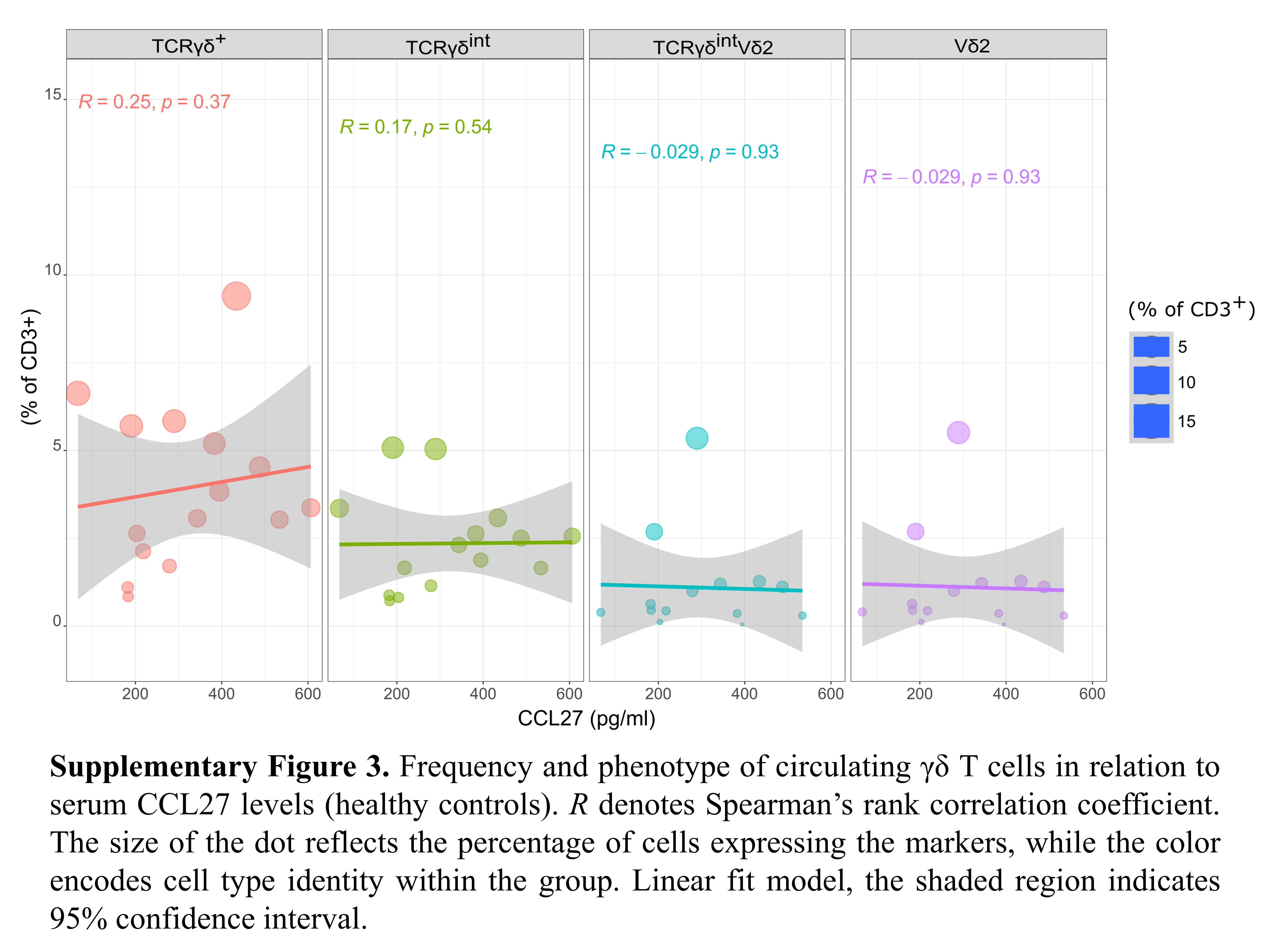

Supplement: Supplementary file 3 [file Image_3.tif]

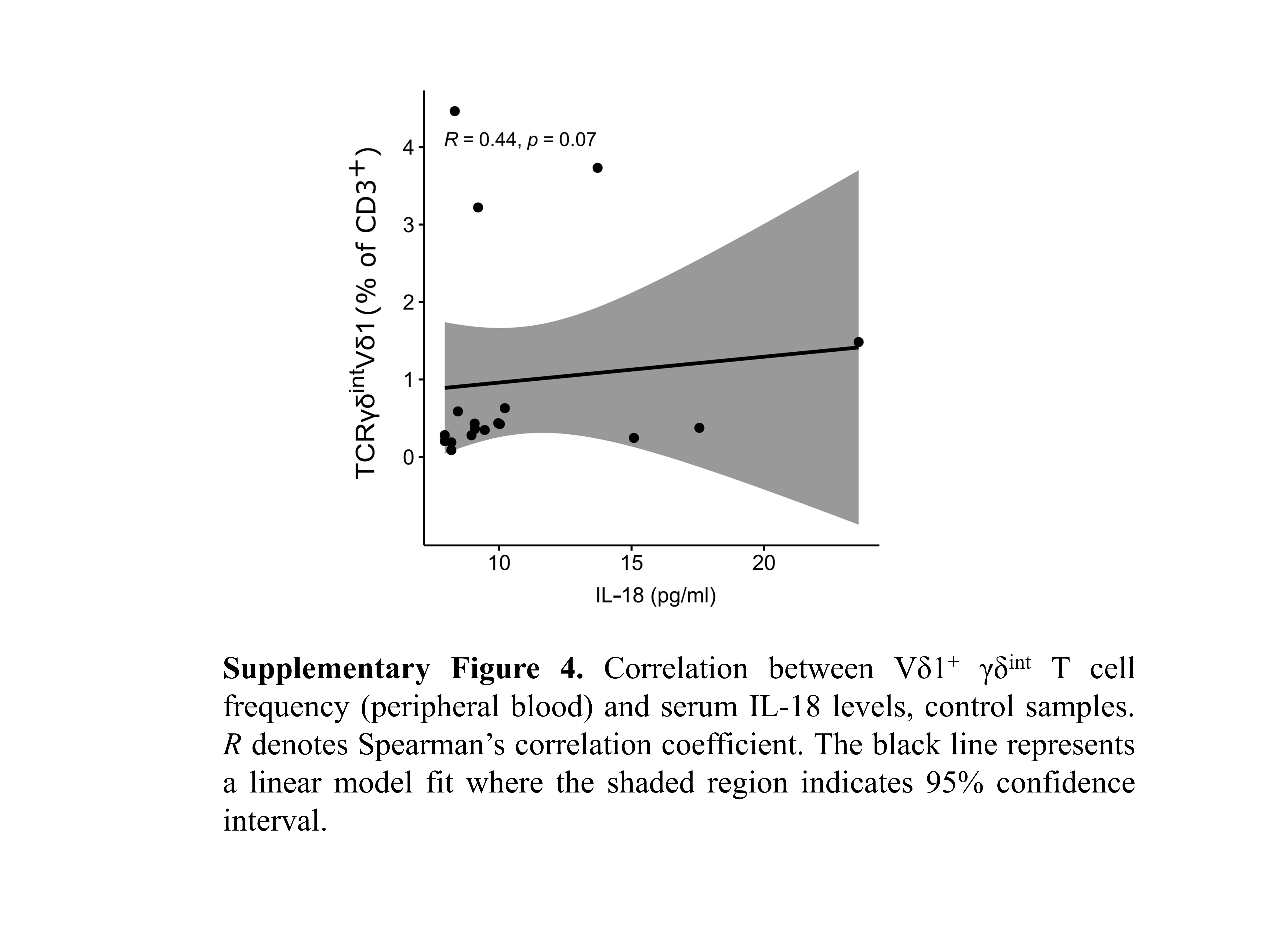

Supplement: Supplementary file 4 [file Image_4.tif]
