# Supplementary material for: Differential Skewing of Circulating MR1-Restricted and γδ T Cells in Human Psoriasis Vulgaris
Source: Front Immunol. 2020 Dec 3;11:572924. doi: 10.3389/fimmu.2020.572924 (PMC7744298; doi:10.3389/fimmu.2020.572924)
Supplement: Supplementary file 5 [file Table_1.docx]

Supplementary Table 1. TaqMan assays of investigated genes.

| Assay ID | Gene abbreviation | Gene name | GenBank accession number | Amplicon size (bp) |
| --- | --- | --- | --- | --- |
| Hs00232313_m1 | PLZF (ZBTB16) | Promyelocytic Leukemia Zinc Finger Protein; Zinc Finger And BTB Domain Containing 16 | NM_001018011.1 | 67 |
| Hs01091094_m1 | RUNX3 | Runt-related transcription factor 3 | NM_001018011.2 | 108 |
| Hs01076112_m1 | RORC | RAR Related Orphan Receptor C | NM_001018011.3 | 62 |
| Hs00706455_s1 | CCR10 | C-C Motif Chemokine Receptor 10 | NM_001018011.4 | 147 |
| Hs00171121_m1 | CCR6 | C-C Motif Chemokine Receptor 6 | NM_001018011.5 | 63 |
| Hs00187256_m1 | IL-18R | Interleukin-18 receptor | NM_001018011.7 | 67 |
| Hs01060665_g1 | ACTB (actin) | Actin beta | NM_001018011.8 | 63 |
| Hs00894392_m1 | TBX21 | T-box transcription factor 21 | NM_001018011.9 | 119 |
| Hs00172872_m1 | EOMES | Eomesodermin | NM_001018011.10 | 81 |
| Hs99999910_m1 | TBP | TATA box binding protein | NM_001018011.11 | 127 |
