# Supplementary material for: Differential Skewing of Circulating MR1-Restricted and γδ T Cells in Human Psoriasis Vulgaris
Source: Front Immunol. 2020 Dec 3;11:572924. doi: 10.3389/fimmu.2020.572924 (PMC7744298; doi:10.3389/fimmu.2020.572924)
Supplement: Supplementary file 6 [file Table_2.docx]

**Supplementary Table 2. Peripheral blood frequencies of CD3^+^γδTCR^high^, CD3^+^γδTCR^int^ T cells (cryopreserved PBMC, % CD3^+^), and their respective TCRδ subsets (median, interquartile range) in healthy controls (n=18) and PV patients (n=16).**

| **CD3^+^ γδTCR^+^** | | |  |
| --- | --- | --- | --- |
|  | Controls (%) | PV (%) | Mann-Whitney P |
| **CD3^+^γδTCR^high^** | | | |
| Vδ1^+^Vδ2^-^ | 0.653 (0.215-1.85) | 0.745 (0.376-1.143) | 0.931 |
| Vδ1^-^Vδ2^+^ | 0.003 (0-0.01) | 0.007 (0.002-0.02) | 0.177 |
| Vδ1^-^Vδ2^-^ | 0.149 (0.056-0.274) | 0.114 (0.051-0.225) | 0.593 |
| **CD3^+^γδTCR^int^** | | |  |
| Vδ1+Vδ2- | 0.37 (0.24-0.81) | 0.311 (0.239-0.85) | 0.666 |
| Vδ1-Vδ2+ | 0.832 (0.382-1.216) | 0.37 (0.238-1.35) | 0.293 |
| Vδ1-Vδ2- | 0.48 (0.23-0.9) | 0.813 (0.3-1.18) | 0.262 |

Data are presented as medians with interquartile range (IQR**).**
