# Supplementary material for: Differential Skewing of Circulating MR1-Restricted and γδ T Cells in Human Psoriasis Vulgaris
Source: Front Immunol. 2020 Dec 3;11:572924. doi: 10.3389/fimmu.2020.572924 (PMC7744298; doi:10.3389/fimmu.2020.572924)
Supplement: Supplementary file 7 [file Table_3.docx]

**Supplementary Table 3. Peripheral blood proportions of γδTCR^high^, γδTCR^int^ T cells (cryopreserved PBMC, % CD3^+^γδTCR^+^), and their respective TCRδ subsets (median, interquartile range) in healthy controls (n=18) and PV patients (n=16).**

| **CD3^+^γδTCR^+^** | | |  |
| --- | --- | --- | --- |
|  | Controls (%) | PV (%) | Mann-Whitney P |
| **CD3^+^γδTCR^high^** | | | |
| Vδ1^+^Vδ2^-^ | 22.8 (9.4-34.5) | 27 (13.3-30) | 0.5 |
| Vδ1^-^Vδ2^+^ | 0.09 (0-0.35) | 0.24 (0.09-0.56) | 0.104 |
| Vδ1^-^Vδ2^-^ | 4.1 (1.6-7.3) | 3.6 (1.8-6.7) | 0.93 |
| **CD3^+^γδTCR^int^** | | |  |
| Vδ1+Vδ2- | 13.1 (9-30.7) | 17.3 (6.3-31.6) | 0.77 |
| Vδ1-Vδ2+ | 29 (11.2-52) | 16.6 (8-34.6) | 0.221 |
| Vδ1-Vδ2- | 16.9 (8.5-24.9) | 21.4 (16.5-28.4) | 0.116 |

Data are presented as medians with interquartile range (IQR).
